# Deep medullary veins score is associated with idiopathic normal pressure hydrocephalus in patients with arteriolosclerotic cerebral small vessel disease

**DOI:** 10.3389/fnins.2026.1854469

**Published:** 2026-08-25

**Authors:** Lijuan Liang, Qiaoling Wu, Jiaxin Chen, Jiaxin Cai, Jing Huang, Qishan He, Kaiyan Feng, Guofang Zeng, Chunwan Chen, Haifeng Huang, Jianming Lei, Xiong Zhang, Shisheng Ye, Hao Li

**Affiliations:** 1 The First School of Clinical Medicine, Guangdong Medical University, Zhanjiang, China; 2 Department of Neurology, Maoming People’s Hospital, Maoming, China; 3 Department of Rehabilitation Medicine, The Third Affiliated Hospital of Sun Yat-sen University, Zhaoqing, China; 4 Department of Integrated Therapy, Maoming People’s Hospital, Maoming, China; 5 Dongguan Chang’an Hospital, Dongguang, China; 6 The First School of Clinical Medicine, Southern Medical University, Guangzhou, China; 7 Department of Neurology, Gaozhou People’s Hospital, Maoming, China; 8 Leliu Hospital Affiliated with Shunde Hospital of Guangzhou University of Chinese Medicine, Foshan, China

**Keywords:** arteriolosclerotic cerebral small vessel disease, deep medullary veins, idiopathic normal pressure hydrocephalus, imaging biomarker, white matter hyperintensity

## Abstract

**Objective:**

This study aimed to investigate the association between deep medullary veins (DMVs) score and comorbid idiopathic normal pressure hydrocephalus (iNPH) in patients with arteriolosclerotic cerebral small vessel disease (aCSVD), and to analyze its role as an independent associated factor.

**Methods:**

A total of 80 patients with aCSVD, 40 patients with arteriolosclerotic cerebral small vessel disease comorbid with idiopathic normal pressure hydrocephalus (aCSVD-iNPH), and 30 healthy controls were enrolled. All participants underwent 3.0-T brain MRI to assess DMVs score, periventricular white matter hyperintensity (PVWMH) volume, deep white matter hyperintensity (DWMH) volume, lacunes, cerebral microbleeds, and total CSVD burden score. Clinical and laboratory data were also collected. Multivariate logistic regression was used to identify independent associated factors, and receiver operating characteristic (ROC) curves were employed to evaluate the discriminative ability of the identified factors for group allocation.

**Results:**

The DMVs score in the aCSVD-iNPH group (15.29 ± 3.67) was significantly higher than that in the aCSVD group (11.73 ± 2.83) and the healthy control group (11.57 ± 1.79) (*P* < 0.001). Multivariate logistic regression showed that DMVs score (OR = 1.499, 95%CI: 1.212–1.855, *P* < 0.001) and PVWMH volume (OR = 1.070, 95%CI: 1.018–1.124,*P* = 0.007) were independent associated factors for aCSVD-iNPH. The combined model incorporating both DMVs score and PVWMH volume achieved a higher area under the ROC curve (0.863) than either indicator alone, with a sensitivity of 72.5% and a specificity of 90.0%.

**Conclusion:**

Elevated DMVs score is associated with comorbid iNPH in aCSVD patients and serves as an independent associated factor. The combination of DMVs score and PVWMH volume may represent a promising imaging biomarker for identifying individuals with aCSVD-iNPH, pending prospective validation.

## 1 Introduction

Cerebral small vessel disease (CSVD) encompasses a spectrum of vascular disorders affecting the small arteries, arterioles, capillaries, and venules of the brain (Yang et al., 2022). Among its subtypes, arteriolosclerotic CSVD (aCSVD) is the most prevalent, primarily driven by vascular risk factors such as hypertension, and is pathologically characterized by hyaline degeneration and fibrinoid necrosis of the small arterial wall. Radiologically, aCSVD manifests as white matter hyperintensities (WMH), lacunar infarcts (LI), and cerebral microbleeds (CMBs), and is closely associated with cognitive impairment, gait disturbance, and vascular dementia (Chou et al., 2021; Goeldlin et al., 2022; Tchuisseu-Kwangoua et al., 2025; Ye et al., 2025).

Idiopathic normal pressure hydrocephalus (iNPH) is a neurodegenerative disorder resulting from cerebrospinal fluid (CSF) dynamics imbalance. It typically presents with the clinical trial of gait impairment, cognitive decline, and urinary incontinence, along with imaging evidence of ventriculomegaly (Evans index ≥ 0.3) and normal CSF pressure on lumbar puncture (Hydrocephalus Study Group of the Neurodegenerative Diseases Committee of the Chinese Society of Microcirculation, Geriatrics Branch of Chinese Medical Association, and Beijing Society of Neurodegenerative Diseases, 2022; Panciani et al., 2025). With population aging and the widespread use of neuroimaging, the comorbidity of aCSVD and iNPH (hereafter referred to as aCSVD-iNPH) has garnered increasing attention. These two conditions exhibit considerable clinical overlap (Cai et al., 2025; Chen et al., 2024; Panciani et al., 2025), and patients with iNPH frequently show multiple CSVD imaging markers (Johansson et al., 2016), A prospective cohort study reported that 78% of iNPH patients present with at least one CSVD imaging marker, and that the total CSVD burden is closely related to motor, cognitive, and prognostic outcomes (Cai et al., 2025), suggesting potential shared pathological mechanisms that may mutually exacerbate neurological dysfunction.

Deep medullary veins (DMVs) are critical structures for maintaining white matter perfusion homeostasis and clearing metabolic waste (Taoka et al., 2017). They participate in the drainage of β-amyloid, tau protein, and other substances, regulate CSF-interstitial fluid exchange, and influence glymphatic system function (Han et al., 2024; Khalatbari et al., 2021; Ma and Han, 2026; Shlobin et al., 2024; Toader et al., 2023; Zhang et al., 2021). The integrity of DMVs can be semi-quantitatively assessed using susceptibility-weighted imaging (SWI) (Zhang et al., 2025). Recent studies have confirmed that the DMVs score is significantly associated with WMH, LI, CMBs, Enlarged perivascular spaces (EPVS), and total CSVD burden, positioning it as a novel imaging marker of CSVD and a correlate of cognitive function and stroke outcomes (Chen et al., 2020; Hong et al., 2025; Zhang et al., 2024; Zhang et al., 2025). However, the role of DMVs as a structural hub connecting venous drainage and cerebral small vessel pathology (Han et al., 2024; Lu et al., 2025; Yin et al., 2026; Zhang et al., 2021; Zhang et al., 2025) in the context of aCSVD-iNPH remains unclear. Studies on the relationship between DMVs score and iNPH are extremely limited. A previous study on CSVD combined with iNPH suggested that DMVs might serve as a potential imaging marker for disease severity in patients with CSVD-iNPH, The possible mechanisms involve endothelial and pericyte dysfunction, as well as impairment of cerebrospinal fluid reflux and absorption (Liu et al., 2020). However, due to the lack of a control group in that study, it remains unclear whether there is a difference in DMVs scores between patients with aCSVD-iNPH and those with aCSVD alone, nor whether the DMVs score can serve as an independent factor associated with aCSVD-iNPH.

This study aims to investigate the correlations between the DMVs score and iNPH imaging features as well as CSVD imaging markers, and the potential value of the DMVs score in identifying iNPH comorbidity in patients with aCSVD.

## 2 Materials and methods

This study employed a retrospective case-control design and was conducted in strict accordance with the Declaration of Helsinki. The study population was derived from a prospective clinical registry cohort established at our center since 2021 (Guangdong Provincial Science and Technology Innovation Strategy Special Fund, Project No. 2021S0025), which applied a unified structured data collection protocol for baseline assessment and regular postoperative follow-up in all patients with suspected iNPH. This prospective cohort was approved by the Ethics Committee (Approval No. PJ2022MI-K007-01). In the present retrospective analysis, we used data from this prospectively maintained database. The use of these data for the retrospective analysis was also approved by the Medical Ethics Committee of Maoming People’s Hospital (Approval No. PJ2026MI-K013-01). The requirement for additional informed consent specifically for this retrospective analysis was waived, as all data were anonymized and the study posed no more than minimal risk to participants. This waiver was granted by the ethics committee based on the criteria of minimal risk and the impracticality of re-contacting all participants.

### 2.1 Participants

This case-control study enrolled 120 consecutive patients with aCSVD who attended the Department of Neurology, Maoming People’s Hospital between January 2022 and September 2025. Among them, 80 were assigned to the aCSVD group and 40 to the aCSVD-iNPH group. Additionally, 30 elderly individuals without neurological diseases during the same period were recruited as the healthy control (HC) group. A total of 150 subjects were finally included in the analysis.

#### 2.1.1 Inclusion and exclusion criteria for aCSVD

Inclusion criteria were as follows (Hu et al., 2021): (1) Age ≥ 60 years; (2) presence of at least one of the following atherosclerotic vascular risk factors: smoking, body mass index > 28 kg/m^2^, hypertension, diabetes, impaired fasting glucose or abnormal glucose tolerance, hyperlipidemia, hyperhomocysteinemia, coronary heart disease, or symptomatic stroke; (3) presence of microbleeds in the brainstem, cerebellar dentate nucleus, or basal ganglia on SWI, with or without additional microbleeds in the cerebellar or cerebral lobes.

Exclusion criteria were as follows: (1) cerebral trauma; (2) intracranial space-occupying lesions; (3) central nervous system lesions secondary to infectious, metabolic, immunological, toxic, or neoplastic causes; (4) ischemic stroke due to large cerebral artery occlusion or cardioembolism; (5) severe intracranial arterial stenosis potentially altering cerebral hemodynamics; and (6) intracerebral hemorrhage.

#### 2.1.2 Inclusion and exclusion criteria for iNPH

Inclusion criteria were as follows (Hydrocephalus Study Group of the Neurodegenerative Diseases Committee of the Chinese Society of Microcirculation, Geriatrics Branch of Chinese Medical Association, and Beijing Society of Neurodegenerative Diseases, 2022; Nakajima et al., 2021): (1) Age ≥ 60 years; (2) Presence of at least one of the classic iNPH triad symptoms, including gait instability, cognitive impairment, or urinary incontinence; (3) Cranial MRI showing hydrocephalus with an Evans index ≥ 0.3; (4) Cerebrospinal fluid (CSF) pressure ≤ 180 mmH_2_O; (5) Presence of at least one of the following characteristics: (a) Gait disorder accompanied by neuroimaging features of narrowed cerebral sulci and subarachnoid spaces over the convexity or midline surface of the brain; (b) Symptomatic improvement after a CSF drainage test; (6) The above clinical symptoms cannot be fully explained by other neurological or non-neurological diseases.

Exclusion criteria were as follows: Secondary hydrocephalus due to any of the following causes: intracerebral hemorrhage, subarachnoid hemorrhage, intracranial space-occupying lesions, craniocerebral trauma, intracranial inflammation, neurosyphilis, tumors, congenital hydrocephalus, or aqueductal stenosis (narrow aqueduct of Sylvius).

All 40 iNPH patients were diagnosed according to the established iNPH criteria. To mitigate selection bias in the aCSVD group, patients were enrolled consecutively, and stringent inclusion/exclusion criteria were applied, although the inherent limitations of a retrospective design cannot be entirely eliminated.

### 2.2 MRI protocol and parameters

All MRI examinations were performed on a 3.0 T superconducting scanner (MAGNETOM Skyra, Siemens). The standardized imaging protocol comprised the following sequences:Axial T1 FLAIR-weighted imaging-Key parameters: TR/TE = 1,750/24 ms, echo train length (ETL) = 10, bandwidth (BW) = 41.67 kHz, acquisition matrix = 320 × 224, field of view (FOV) = 240 × 240 mm^2^, slice thickness/gap = 5/1 mm, number of excitations (NEX) = 1.Axial T2 PROPELLER (FrFSE) sequence -Settings: TR/TE = 5,727/93 ms, ETL = 32, BW = 83.3 kHz, matrix size = 512 × 512, FOV = 240 × 240 mm^2^, slice thickness/gap = 5/1 mm, NEX = 1.5.T2 FLAIR sequence-Parameters: TR/TE/TI = 8,400/145/2,100 ms, BW = 83.3 kHz, flip angle = 145°, matrix = 320 × 224, FOV = 240 × 240 mm^2^, slice thickness/gap = 5/1 mm, NEX = 1.Three-dimensional time-of-flight MR angiography (TOF-MRA) -TR/TE = 25/3.4 ms, flip angle = 20°, BW = 41.67 kHz, matrix = 384 × 320, FOV = 200 × 200 mm^2^, isotropic voxel size = 0.8 mmł, NEX = 1.Axial T2-weighted SWAN sequence- TR/TE = 77.3/45 ms, BW = 62.5 kHz, flip angle = 15°, matrix = 384 × 320, slice thickness = 2 mm, NEX = 1.Image interpretation was carried out under blinded conditions: two experienced neurologists independently reviewed all scans, and all imaging data were anonymized and labeled only with identification numbers. For the lesion counts of LI, CMBs, and EPVS, the average of the counts from the two evaluators was used for statistical analysis. For semi-quantitative visual scoring indices such as the Fazekas score for white matter hyperintensities, the total cerebral small vessel disease burden score, and the DMVs score, Cohen’s kappa coefficient was used to assess inter-rater reliability. The results showed that the kappa value for the DMVs score was 0.825, for the periventricular WMH Fazekas score was 0.933, and for the deep WMH Fazekas score was 0.946, all exceeding 0.81, indicating excellent inter-rater agreement. If there was disagreement between the two readers, they jointly re-evaluated the images and reached a consensus to determine the final score.

**FIGURE 1:**
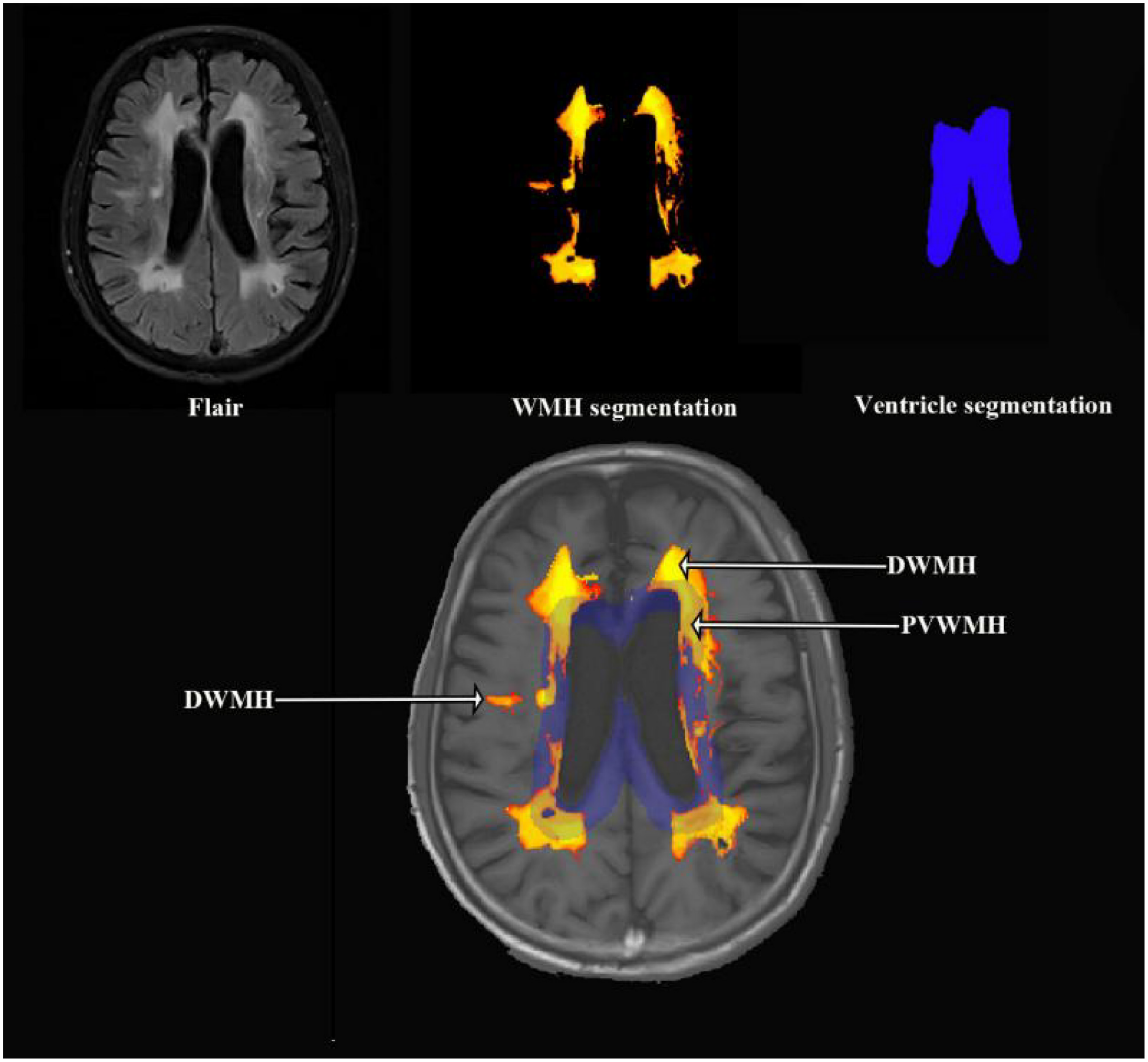
Example of WMH volume calculation chart. Adapted from Ye et al. (2025).

Based on the SPM12 platform, the LGA algorithm of the LST toolbox (version 3.0) was used to fuse the registered T1 and FLAIR images, and a probability threshold of 0.5 was applied to generate the WMH volume. Subsequently, FSL was used to extract the lateral ventricle mask, and a distance of 10 mm from the ventricular edge was set to define the periventricular white matter hyperintensities (PVWMH). The PVWMH volume was obtained by multiplying this mask with the WMH probability map. The DWMH volume was calculated by subtracting the PVWMH volume from the total WMH volume, Quality assurance involved a two-step visual inspection of both the raw and preprocessed MRI datasets to confirm data fidelity. Scans exhibiting evident oversegmentation, motion or susceptibility artifacts, or any segmentation inaccuracies were disqualified from subsequent statistical analyses (Ye et al., 2025), an example is shown in Figure 1 (Cedres et al., 2020). The overall CSVD burden was evaluated according to the STRIVE criteria (Wardlaw et al., 2013). The CSVD burden score (total SVD score) was derived from four MRI markers: LI, WMH, EPVS, and CMBs, with a maximum total score of 4 (Staals et al., 2014). One point was assigned for each of the following: presence of ≥ 1 lacune, presence of ≥ 1 CMB, moderate-to-severe (grade 2–4) EPVS in the basal ganglia, PVWMH of Fazekas grade 3, or DWMH of Fazekas grade 2–3.

**FIGURE 2:**
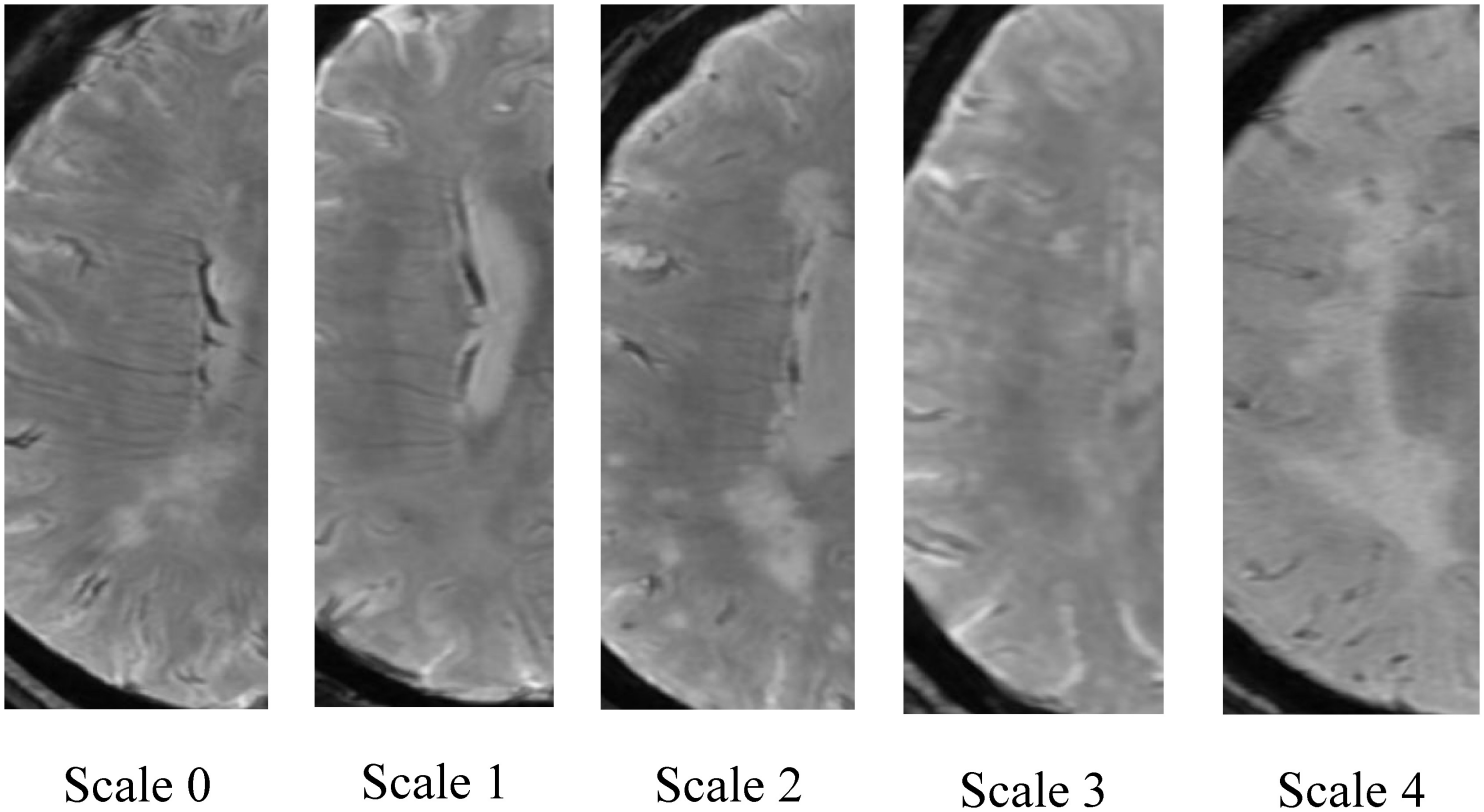
DMVs grading scale.

The DMVs score was assessed using a 0–4 regional grading system (Chen et al., 2020; Figure 2): Scale 0 = continuous and homogeneous DMVs signal within the region of interest (ROI);Scale 1 = discontinuous or inhomogeneous signal in ≤ half of the ROI; Scale 2 = discontinuous or inhomogeneous signal in > half of the ROI; Scale 3 = blurred DMVs contour with poor delineation within the ROI; Scale 4 = no visible DMVs in the ROI. For each hemisphere, the same slice was scored, and the average of the two sides served as the grade for that slice. The total DMVs score (maximum 20 points) was obtained by summing the scores from five slices.

### 2.3 Data collection

Demographic characteristics, vascular risk factors, laboratory results, and neuroimaging data were collected. Demographic data included age and sex. Vascular risk factors included smoking, alcohol use, hypertension, diabetes, and ischemic stroke. Laboratory tests on admission included white blood cell count, neutrophil count, lymphocyte count, monocyte count, red blood cell count, hemoglobin, platelet count, total cholesterol (TC), triglycerides (TG), high-density lipoprotein cholesterol (HDL-C), low-density lipoprotein cholesterol (LDL-C), and homocysteine. All laboratory tests were performed using standard methods.

### 2.4 Statistical analysis

All statistical analyses were performed using SPSS version 27.0. Continuous variables following a normal distribution were presented as mean ± standard deviation (Mean ± SD), while non-normally distributed continuous variables were expressed as median (interquartile range). Categorical variables were summarized as frequencies (percentages). Between-group comparisons were conducted based on data type and distribution characteristics. For categorical variables, the Pearson chi-square test was applied. For normally distributed continuous variables, the independent-samples *t*-test was used for two-group comparisons. For non-normally distributed continuous or ordinal variables, the Mann-Whitney U test was employed for two-group comparisons, and the Kruskal-Wallis test for multi-group comparisons. Variables that were clinically meaningful or had a *P* < 0.05 in univariate analysis were entered into a multivariate binary logistic regression model to identify independent predictors of iNPH among patients with aCSVD. Receiver operating characteristic (ROC) curves were then used to evaluate the discriminative ability of each independent factor and their combination. The area under the curve (AUC), optimal cutoff value (determined by maximizing the Youden index), sensitivity, and specificity were calculated. A two-tailed *P* < 0.05 was considered statistically significant. Multicollinearity among the variables in the multivariate model was assessed using variance inflation factors (VIFs) and tolerance values, with VIF > 10 indicating significant collinearity.

## 3 Results

### 3.1 Comparison of baseline characteristics and DMVs scores between groups

There were no significant differences in age, history of diabetes, or alcohol use among the three groups (*P* > 0.05). The proportion of males in the aCSVD-iNPH group (87.50%) was significantly higher than that in the aCSVD group (66.25%) (*P* < 0.05), while there were no significant differences in hypertension, diabetes, smoking, alcohol use, or history of stroke between the two aCSVD groups (*P* > 0.05). The DMVs score in the aCSVD-iNPH group (15.29 ± 3.67) was significantly higher than that in the aCSVD group (11.73 ± 2.83) (*P* < 0.001) and also higher than that in the HC group (11.57 ± 1.79) (*P* < 0.001), whereas there was no significant difference in DMVs score between the aCSVD group and the HC group (*P* = 0.792) (Table 1).

**TABLE 1:** Comparison of general information and DMVs scores.

Variable	Acsvd (*n* = 80)	aCSVD-iNPH (*n* = 40)	HC (*n* = 30)	*X*^2^/*F*	*P*
Age (years)	66.89 ± 9.36^#^	70.80 ± 8.25	69.17 ± 6.07	2.976	0.054
Male (%)	53 (66.25)^#^ *	35 (87.50)***	13 (43.33)	15.295	< 0.001
History of hypertension (%)	67 (83.75)***	29 (72.50)***	7 (23.33)	24.402	< 0.001
History of diabetes (%)	25 (31.25)	10 (25)	4 (13.33)	37.388	0.160
History of smoking (%)	13 (16.25)*	8 (20)*	0 (0)	6.416	0.040
History of alcohol use (%)	5 (6.25)	1 (2.5)	0 (0)	2.539	0.281
History of stroke (%)	44 (55)***	24 (60)***	0 (0)	31.367	< 0.001
DMVs score	11.73 ± 2.83^###^	15.29 ± 3.67***	11.57 ± 1.79	22.344	< 0.001

aCSVD vs. HC: *(*P* < 0.05), ***(*P <* 0.001); aCSVD-iNPH vs. HC: *(*P* < 0.05), ***(*P* < 0.001); aCSVD-iNPH vs. aCSVD: ^#^(*P* < 0.05), ^###^(*P* < 0.001).

### 3.2 Comparison of imaging markers between the aCSVD-iNPH and aCSVD groups

Compared with the aCSVD group, the aCSVD-iNPH group showed a more severe burden of cerebral small vessel disease across multiple imaging markers: DMVs score (15.29 ± 3.67 vs. 11.73 ± 2.83, *P* < 0.001), Evans index (0.33 ± 0.04 vs. 0.25 ± 0.03, *P* < 0.001), PVWMH volume [21.66 (13.7, 32.8) vs. 8.85 (3.5, 15.8), *P* < 0.001], DWMH volume [6.52 (3.0, 13.3) vs. 2.32 (0.5, 5.8), *P* < 0.001], number of lacunes [4 (2.3, 7.0) vs. 3 (1.0, 5.8), *P* = 0.022], number of lobar CMBs [9 (3, 17) vs. 4.5 (1, 10), *P* = 0.011], and total CSVD burden score [4 (3, 4) vs. 3 (3, 4), *P* = 0.028] were all significantly higher. No significant differences were found in CMBs of the brainstem, dentate nucleus, cerebellar lobes, basal ganglia, or deep regions, nor in PVS grading of the bilateral basal ganglia or centrum semiovale (*P* > 0.05) (Table 2).

**TABLE 2:** Comparison of imaging markers between the aCSVD—iNPH group and the aCSVD group.

Variable	aCSVD-iNPH (*n* = 40)	aCSVD (*n* = 80)	*t/Z*	*P*
DMVs score	15.29 ± 3.67	11.73 ± 2.83	−5.375	< 0.001
EI	0.33 ± 0.04	0.25 ± 0.03	−13.437	< 0.001
PVWMH volume	21.66 (13.7, 32.8)	8.85 (3.5, 15.8)	−5.150	< 0.001
DWMH volume	6.52 (3.0, 13.3)	2.32 (0.5, 5.8)	−5.133	< 0.001
LI	4 (2.3, 7.0)	3 (1.0, 5.8)	−2.282	0.022
BS-CMBs	1.5 (0, 4.8)	2 (0, 4)	−0.037	0.971
DN-CMBs	0.5 (0, 2)	0 (0, 2)	−0.970	0.332
CL-CMBs	0 (0, 2.8)	0 (0, 1)	−1.650	0.099
BG CMBs	11.5 (4.3, 20.5)	8 (3, 15)	−1.725	0.085
Lobar CMBs	9 (3, 17)	4.5 (1, 10)	−2.557	0.011
Deep CMBs	15 (6, 29.5)	10.5 (4.3, 20.8)	−1.373	0.170
L-BG-PVS	2 (1, 3)	2 (1, 3)	−0.059	0.953
R-BG-PVS	2 (1, 3)	2 (1, 3)	−0.554	0.580
L-CSO-PVS	0 (0, 1)	0 (0, 1)	−1.724	0.085
R-CSO-PVS	0 (0, 1)	0 (0, 0)	−1.450	0.147
Burden score	4 (3, 4)	3 (3, 4)	−2.201	0.028

DMVs score, deep medullary veins score; EI, Evans index; PVWMH volume, periventricular white matter hyperintensity volume; DWMH volume, deep white matter hyperintensity volume; LI, lacunes; BS-CMBs, brainstem cerebral microbleeds; DN-CMBs, dentate nucleus cerebral microbleeds; CL-CMBs, cerebellar lobar cerebral microbleeds; BG CMBs, basal ganglia cerebral microbleeds; Lobar CMBs, lobar cerebral microbleeds; Deep CMBs, deep cerebral microbleeds; L-BG-PVS, left basal ganglia perivascular spaces; R-BG-PVS, right basal ganglia perivascular spaces; L-CSO-PVS, left centrum semiovale perivascular spaces; R-CSO-PVS, right centrum semiovale perivascular spaces; Burden score, cerebral small vessel disease burden score; Deep CMBs = (dentate nucleus + brainstem + basal ganglia) CMBs.

### 3.3 Comparison of laboratory parameters between the aCSVD-iNPH and aCSVD groups

Regarding laboratory parameters, the aCSVD-iNPH group exhibited significantly lower red blood cell count (4.44 ± 0.64 vs. 4.73 ± 0.66,*P* = 0.020), hemoglobin (130.44 ± 16.18 vs. 138.73 ± 15.88, *P* = 0.008), and LDL-C (2.63 ± 0.72 vs. 3.01 ± 0.93, *P* = 0.025), whereas creatinine [100.80 (83.8–125.3) vs. 86.90 (72.0–98.0), *P* = 0.002], blood urea nitrogen [6.24 (4.6–7.5) vs. 5.28 (4.1–6.0), *P* = 0.006], and homocysteine [18.49 (15.0–20.7) vs. 16.12 (12.5–17.5), *P* = 0.009] were significantly higher. Total cholesterol showed a marginal difference between the two groups [4.31 (3.7–5.2) vs. 4.87 (3.9–5.6), *P* = 0.050]. Other parameters, including white blood cell count, NLR, SII, platelet count, ALT, uric acid, triglycerides, and HDL-C, showed no statistically significant differences (*P* > 0.05) (Table 3).

**TABLE 3:** Comparison of hematological markers between the aCSVD-iNPH group and the aCSVD group.

Variable	aCSVD-iNPH (*n* = 40)	aCSVD (*n* = 80)	*t/Z*	*P*
WBC ( × 10^9/L)	7.82 (6.4, 9.1)	7.46 (6.4, 9.2)	−0.501	0.616
NLR (%)	3.31 (2.0, 5.4)	3.08 (2.2, 4.1)	−0.607	0.544
SII (10^9 /L)	1809.63 (1197.8, 2475.3)	2015.56 (1300.5, 2638.8)	−0.440	0.660
RBC (10^12/L)	4.44 ± 0.64	4.73 ± 0.66	2.355	0.020
Hb (g/L)	130.44 ± 16.18	138.73 ± 15.88	2.677	0.008
PLT (10^9/L)	231.68 ± 62.73	235.86 ± 61.40	0.349	0.728
ALT (mmol/L)	18.30 (12.7, 25.5)	17.50 (12.9, 22.9)	−0.109	0.914
Cr (μmol/L)	100.80 (83.8, 125.3)	86.90 (72.0, 98.0)	−3.110	0.002
UA (μmol/L)	374.25 (306.0, 431.3)	362.81 (310.0, 398.8)	−0.713	0.476
BUN (mmol/L)	6.24 (4.6, 7.5)	5.28 (4.1, 6.0)	−2.749	0.006
HCY (μmol/L)	18.49 (15.0, 20.7)	16.12 (12.5, 17.5)	−2.611	0.009
TG (mmol/L)	1.15 (0.8, 1.5)	1.28 (0.8, 2.0)	−0.715	0.474
TC (mmol/L)	4.31 (3.7, 5.2)	4.87 (3.9, 5.6)	−1.960	0.050
HDL-C (mmol/L)	1.14 (1.0, 1.3)	1.19 (1.0, 1.4)	−0.754	0.451
LDL-C (mmol/L)	2.63 ± 0.72	3.01 ± 0.93	2.273	0.025

WBC, white blood cell count; NLR, neutrophil-to-lymphocyte ratio; SII, systemic immune-inflammation index; RBC, red blood cell count; Hb, hemoglobin; PLT, platelet count; ALT, alanine aminotransferase; Cr, creatinine; UA, uric acid; BUN, blood urea nitrogen; HCY, homocysteine; TG, triglycerides; TC, total cholesterol; HDL-C, high-density lipoprotein cholesterol; LDL-C, low-density lipoprotein cholesterol.

### 3.4 Analysis of independent risk factors for aCSVD-iNPH

Multivariate binary logistic regression analysis showed that DMVs score (OR = 1.499, 95% CI: 1.212∼1.855, *P* < 0.001) and PVWMH volume (OR = 1.070, 95% CI: 1.018∼1.124, *P* = 0.007) were independent factors associated with aCSVD-iNPH (Table 4). Collinearity assessment of the model revealed that all VIF values (range: 1.092–1.976) were below 2.0 and all tolerance values were > 0.5, far within the commonly accepted limits (VIF < 10, tolerance > 0.1), confirming that multicollinearity was not a concern and that each variable contributed largely unique information to the model.

**TABLE 4:** Multivariate logistic regression analysis of influencing factors for the aCSVD-iNPH Group.

Variable	β	OR	95% CI	*P*
Age (years)	0.039	1.039	0.971 ∼ 1.113	0.270
Male(%)	−1.444	0.236	0.041 ∼ 1.353	0.105
LDL-C (mmol/L)	−0.290	0.748	0.340 ∼ 1.645	0.470
Hb (g/L)	−0.003	0.997	0.955 ∼ 1.040	0.883
BUN (mmol/L)	0.092	1.096	0.887 ∼ 1.356	0.395
Cr (μmol/L)	−0.000	1.000	0.988 ∼ 1.012	0.987
HCY (mmol/L)	0.023	1.023	0.926 ∼ 1.130	0.655
DMVs score	0.405	1.499	1.212 ∼ 1.855	< 0.001
DWMH volume	0.027	1.027	0.992 ∼ 1.064	0.135
PVWMH volume	0.067	1.070	1.018 ∼ 1.124	0.007
LI	0.063	1.065	0.941 ∼ 1.204	0.318
Lobar CMBs	0.018	1.018	0.984 ∼ 1.053	0.297
Burden score	0.263	1.300	0.469 ∼ 3.605	0.614

LDL-C, low-density lipoprotein cholesterol; Hb, hemoglobin; BUN, blood urea nitrogen; Cr, creatinine; HCY, homocysteine; DMVs score, Deep medullary veins score; DWMH volume, deep white matter hyperintensity volume; PVWMH volume, periventricular white matter hyperintensity volume; LI, lacunes; Lobar CMBs, lobar cerebral microbleeds; Burden score, cerebral small vessel disease burden score.

### 3.5 The ability of DMVs score and PVWMH volume to identify iNPH

**FIGURE 3:**
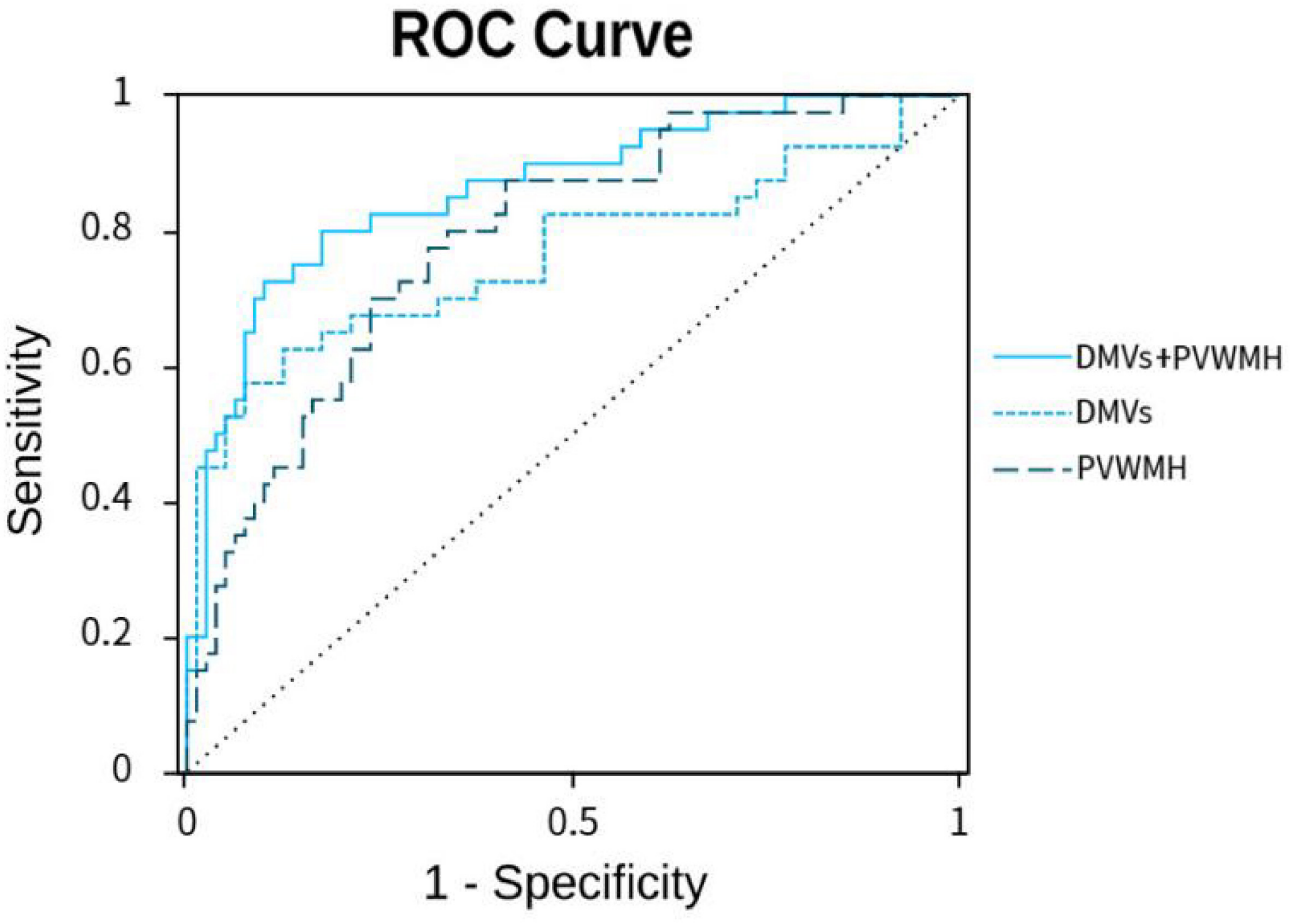
ROC curves of DMVs score and combined PVWMH volume for predicting the occurrence of iNPH.

ROC curve analysis was performed for DMVs score and PVWMH volume, respectively. Both the DMVs score and PVWMH volume showed modest discriminative ability for iNPH. The AUC for DMVs score was 0.779 (95% CI: 0.680∼0.879), with an optimal diagnostic cutoff of 14.5, yielding a sensitivity of 62.5% and a specificity of 87.5%. The AUC for PVWMH volume was 0.788 (95% CI: 0.704∼0.872), with an optimal cutoff of 13.093 mL, corresponding to a sensitivity of 77.5% and a specificity of 68.8%. When DMVs score and PVWMH volume were combined, the AUC increased to 0.863 (95% CI: 0.792∼0.935), with a sensitivity of 72.5% and a specificity of 90.0% (Figure 3).

### 3.6 Comparison between high DMVs score group and low DMVs score group

Using the optimal cutoff value of 14.5 for the DMVs score, 120 patients (including those with aCSVD and aCSVD-iNPH) were divided into two groups: a low-score group (80 cases) and a high-score group (40 cases). Intergroup comparisons revealed that the high-score group had a significantly higher Evans index (0.30 ± 0.05 vs. 0.26 ± 0.05,*P* < 0.001), blood urea nitrogen [6.24 (5.3–7.5) vs. 5.10 (4.2–6.0), *P* = 0.001], creatinine [95.85 (84.4–126.2) vs. 86.55 (73.1–101.8),*P* = 0.008], and uric acid [379 (330.5–452.3) vs. 351 (306.0–387.8), *P* = 0.018], as well as a significantly lower red blood cell count (4.37 ± 0.64 vs. 4.77 ± 0.64, *P* = 0.001), hemoglobin concentration (128.04 ± 15.62 vs. 139.93 ± 15.38, *P* < 0.001), and triglyceride [1.03 (0.7–1.4) vs. 1.37 (0.9–2.1), *P* = 0.014]. No statistically significant differences were observed between the two groups for other variables, including age, neutrophil-to-lymphocyte ratio (NLR), systemic immune-inflammation index (SII), platelet count, alanine aminotransferase, total cholesterol, and homocysteine (*P* > 0.05) (Table 5).

**TABLE 5:** Comparison between the low-score group and the high-score group.

Variable	Low-score (*n* = 80)	High-score (*n* = 40)	*t/Z*	*P*
Evans index	0.26 ± 0.05	0.30 ± 0.05	−4.42	< 0.001
Age (years)	67.46 ± 9.81	69.65 ± 7.59	−1.236	0.219
NLR(%)	3.01 (2.0, 4.7)	3.35 (2.4, 4.5)	−1.138	0.255
SII (10^9/L)	2060.98 (1260.9, 2595.1)	1874.05 (1097.3, 2475.3)	−0.234	0.815
RBC (10^12/L)	4.77 ± 0.64	4.37 ± 0.64	3.252	0.001
Hb (g/L)	139.93 ± 15.38	128.04 ± 15.62	3.970	< 0.001
LDL-C (mmol/L)	2.94 ± 0.91	2.77 ± 0.81	1.027	0.307
ALT (mmol/L)	18.35 (13.9, 23.4)	15.75 (11.9, 23.3)	−0.894	0.371
TG (mmol/L)	1.37 (0.9, 2.1)	1.03 (0.7, 1.4)	−2.447	0.014
TC (mmol/L)	4.71 (4.0, 5.6)	4.42 (3.6, 5.3)	−1.531	0.126
BUN (mmol/L)	5.10 (4.2, 6.0)	6.24 (5.3, 7.5)	−3.297	0.001
Cr (μmol/L)	86.55 (73.1, 101.8)	95.85 (84.4, 126.2)	−**2**.636	0.008
UA (μmol/L)	351 (306.0, 387.8)	379 (330.5, 452.3)	−2.367	0.018
HCY (μmol/L)	16.32 (13.7, 18.4)	17.17 (12.8, 19.9)	−0.774	0.439

NLR, neutrophil-to-lymphocyte ratio; SII, systemic immune-inflammation index; RBC, red blood cell count; Hb, hemoglobin; PLT, platelet count; LDL-C, low-density lipoprotein cholesterol; ALT, alanine aminotransferase; TG, triglycerides; TC, total cholesterol; BUN, blood urea nitrogen; Cr, creatinine; UA, uric acid; HCY, homocysteine.

Univariate logistic regression (Model 1) showed that patients in the high-score group had a substantially increased risk of iNPH compared with those in the low-score group, with an odds ratio (OR) of 8.755 (95% CI: 3.673–20.872, *P* < 0.001). After multivariable adjustment for potential confounders (red blood cell count, blood urea nitrogen, triglyceride, creatinine, and uric acid) in Model 2, the association remained significant (OR = 7.905, 95% CI: 2.981∼20.966, *P* < 0.001), indicating that an elevated DMVs score is independently associated with an increased risk of iNPH. The model demonstrated good calibration, as reflected by the Hosmer-Lemeshow goodness-of-fit test (*P* = 0.126) (Table 6).

**TABLE 6:** Comparison of iNPH risk between high-score and low-score groups.

DMVs score	Median	iNPH, *n*(%)	Model 1		Model 2	
			OR (95% CI)	*P*	OR (95% CI)	*P*
Low-score (*n* = 80)	11.25	14 (17.50)	8.755 (3.673 ∼ 20.872)	< 0.001	7.905 (2.981 ∼ 20.966)	< 0.001
High-score (*n* = 40)	16.5	26 (65.00)				

## 4 Discussion

This study systematically revealed that compared with aCSVD patients, those with aCSVD-iNPH had significantly higher DMVs scores and PVWMH volumes, both of which were independent factors associated with aCSVD-iNPH, whereas DWMH volume was not retained in the regression model. This finding carries important pathophysiological implications. PVWMH are located in the periventricular region, where venous drainage depends mainly on the DMVs system and is closely linked to cerebrospinal fluid dynamics (Gao et al., 2025; Lahna et al., 2022). In contrast, DWMH are more reflective of deep arterial perfusion abnormalities (Cai et al., 2022; Hannawi et al., 2022). Therefore, the absence of DWMH from the model suggests that venous or periventricular drainage dysfunction may play a more critical role than deep arteriosclerosis in the comorbid state of aCSVD-iNPH. Furthermore, the lack of a significant difference in DMVs score between the aCSVD group and healthy controls may be explained by the predominantly arterial nature of isolated aCSVD, which involves less prominent venous pathology, and the relatively narrow age range (all ≥ 60 years) may have attenuated the potential effect of age on DMVs score (Ao et al., 2021; Li et al., 2026).

Further analysis revealed that the combined model incorporating both DMVs score and PVWMH volume achieved a significantly higher AUC (0.863) than either indicator alone, indicating a synergistic mechanism. DMVs primarily drain the periventricular white matter, where PVWMH are located. Previous studies have shown that confluent PVWMH often distribute along the venous course (Zhang et al., 2021). Damage to DMVs (e.g., venous collagen deposition, luminal stenosis) impairs venous outflow, resulting in elevated local venous pressure, reduced blood flow, altered trans-capillary pressure gradients, and fluid extravasation into the interstitium. These changes promote vasogenic edema (Gao et al., 2025; Zhang et al., 2021) and subsequently accelerate PVWMH progression. Conversely, worsening PVWMH reflects interstitial fluid accumulation and impaired waste clearance, which may further compromise the DMVs microenvironment, thereby creating a positive feedback loop. Studies have confirmed that extracellular free water mediates the association between DMVs score and WMH volume (Lan et al., 2024). Moreover, glymphatic function depends on arterial pulsation, with interstitial fluid and metabolic wastes ultimately drained via the venous system, including DMVs (Xie et al., 2024). DMVs damage can cause downstream glymphatic outflow obstruction, leading to periventricular deposition of toxic proteins, including Aβ (Eliav et al., 2026; Gao et al., 2025). Collectively, these mechanisms explain the synergistic effect of DMVs and PVWMH in aCSVD-iNPH.

The DMVs score findings in the present study are consistent with multiple imaging markers reported in recent research on venous abnormalities. Chen et al. proposed the DMVs score as a novel imaging biomarker for CSVD (Chen et al., 2020). Our study further validates that the DMVs score is significantly elevated in aCSVD-iNPH patients and remains independent of conventional CSVD markers. Zhang et al. employed the DTI-ALPS method to demonstrate that venous injury compromises white matter integrity (Zhang et al., 2021). Our results support this finding and, for the first time, reveal the independent association of the DMVs score with iNPH comorbidity. Lahna et al. observed that venous collagenosis is closely associated with periventricular white matter hyperintensities (Lahna et al., 2022), which aligns with the synergistic effect between the DMVs score and PVWMH volume documented in our study. Additionally, cerebral venous reflux (CVR) has been linked to the pathological evolution of distinct CSVD subtypes (Lee et al., 2023; Tsai et al., 2022). Cerebral venous disruption is a characteristic imaging feature of CSVD, and a reduced DTI-ALPS index correlates strongly with CSVD severity (Zhang et al., 2025). Enlarged perivascular spaces (EPVS) are closely associated with white matter injury and cognitive decline in aCSVD (Cai et al., 2025; Ma and Han, 2026). In summary, evidence from multidimensional markers (including the DMVs score, CVR, venous disruption, DTI-ALPS, and EPVS) converges to support that venous dysfunction (encompassing impaired outflow, compromised drainage, and structural abnormalities) constitutes a key pathological link in aCSVD and its comorbidity with iNPH.

This study further revealed a significant positive correlation between DMVs score and Evans index, consistent with Manchineella et al. (2026), suggesting a bidirectional interaction between DMVs abnormalities and ventriculomegaly. On one hand, ventriculomegaly may mechanically compress adjacent DMVs, leading to venous lumen obstruction (Schreiber et al., 2020; Zhang et al., 2021). On the other hand, DMVs dysfunction promotes interstitial fluid accumulation and elevates local tissue pressure, thereby exacerbating ventriculomegaly (Zhang et al., 2021). Moreover, DMVs injury impairs glymphatic function, hampers the clearance of toxic proteins such as Aβ, and facilitates periventricular tissue damage (Lowe et al., 2026; Wu et al., 2025). The cerebral venous system plays a key role in neurodegeneration (Schreiber et al., 2020), and DMVs are essential for maintaining blood-brain barrier integrity (Liu et al., 2022; Wang et al., 2025; Zhang et al., 2021). An elevated DMVs score may reflect indirect blood-brain barrier damage, with pathological substrates including venous wall collagen deposition, fibrosis, and hyalinosis, culminating in luminal occlusion, venous wall thickening, and increased vascular permeability (Zhang et al., 2021), thus worsening iNPH symptoms (Hakim and Adams, 1965; Keith et al., 2017). In summary, DMVs abnormalities contribute to the pathogenesis of iNPH through three pathways: venous outflow obstruction, blood-brain barrier disruption, and glymphatic dysfunction, potentially serving as a key bridge linking CSVD and iNPH.

Although this study employed semi-quantitative visual DMV rating and automated volumetric WMH measurement to evaluate imaging markers associated with CSVD, we acknowledge that these methods have inherent limitations, including inter-rater variability, dependence on operator expertise, and difficulty in fully capturing the continuous variation in lesion burden. Recently, Zhao et al. developed a deep learning-based automated segmentation method that enables quantitative CSVD diagnosis from multi-sequence MRI, while simultaneously providing precise quantification of its neuroimaging markers (Zhao et al., 2025). Compared with conventional visual rating systems, these automated quantitative approaches offer several distinct advantages: (1) they eliminate inter-rater variability by delivering objective and reproducible measurements; (2) they permit continuous rather than categorical assessment of lesion burden, potentially enhancing sensitivity to subtle pathological changes; (3) they facilitate large-scale multicenter studies by standardizing image analysis across diverse centers and scanner platforms. Looking forward, integrating deep learning methods with DMV evaluation holds promise for further improving the reproducibility and objectivity of venous imaging markers, thereby enabling a more accurate delineation of the relationship between venous dysfunction and the aCSVD-iNPH comorbidity.

This study has several limitations. The single-center retrospective case-control design cannot establish causality, and the relatively small sample size may introduce selection bias, The external validity of the conclusions is limited. The DMVs score is a qualitative visual rating that can be influenced by equipment, MR slice selection, and inter-observer variability; quantitative validation is needed in the future. Due to the difficulty in clinically confirming aCSVD-iNPH, the limited number of confirmed cases, and the challenges in patient enrollment, sex matching could not be achieved. Future multicenter prospective studies with longitudinal follow-up are needed to further validate the association between DMVs score and iNPH comorbidity, as well as its dynamic changes over time.

## 5 Conclusion

Elevated DMVs score is associated with comorbid iNPH in aCSVD patients and serves as an independent associated factor. The combination of DMVs score and PVWMH volume may represent a promising imaging biomarker for identifying individuals at high risk of aCSVD-iNPH, pending prospective validation.

## Data Availability

The raw data supporting the conclusions of this article will be made available by the authors, without undue reservation.
